# Sexual Behavior, App Use, and Venue Comfort During COVID-19: A Global Study of Gay, Bisexual, and Other Men Who Have Sex with Men

**DOI:** 10.1080/00224499.2025.2585071

**Published:** 2025-11-17

**Authors:** Juan C. Jauregui, Chenglin Hong, Alex Garner, Sean Howell, Ian W. Holloway

**Affiliations:** a Department of Social Welfare, UCLA Luskin School of Public Affairs; b School of Social Work, University of Connecticut; c MPact Global; d Hornet Gay Social Network; e School of Nursing, UCLA

## Abstract

Little global research exists exploring the impact of COVID-19 on the sexual behavior of gay, bisexual, and other men who have sex with men (GBMSM). Using data from a global dataset representing GBMSM from 132 countries, this study assessed sexual behavior and comfort attending social and sexual venues (bars, saunas, sex parties) during the pandemic. Data were derived from a cross-sectional survey implemented by Hornet, a popular gay social-networking app, from October to November 2020. A total of 15,499 GBMSM were included in this analysis. Nearly two thirds (64.8%) of the sample had met a sexual partner through a dating/hookup app since the COVID-19 crisis began. GBMSM waiting to attend social and sexual venues until public health officials indicated it was safe, there was a vaccine, or their friends started going were less likely to have an app-based sex partner. Additionally, both HIV-positive status and current PrEP use were associated with higher odds of app-based sexual contact. These findings highlight the dynamic relationship between digital platforms and in-person sexual contexts during public health crises. Integrating harm reduction and sex-positive messaging across both digital and offline spaces is critical, especially during crises that limit access to social connection and affirming spaces.

## Introduction

Since the first case of the severe acute respiratory syndrome coronavirus 2 (SARS-COV-2) was detected in December 2019, the virus spread rapidly, leading to the global COVID-19 pandemic. As of August 2025, there have been over 778 million cases worldwide and over 7 million deaths (Dong et al., 2020). While COVID-19 is no longer considered a global emergency, periodic surges and the emergence of new variants continue to remain a public health challenge (Hohman, 2024). The public health response to COVID-19 differed drastically across the globe with varying degrees of vaccine rollout and “stay-at-home” orders that impacted the spread of the virus (Jain et al., 2022; Ren, 2020). Social and physical distancing guidelines were implemented by public health agencies, which recommended limiting sexual contact with individuals outside the primary household (Meunier et al., 2021).

These guidelines posed distinct challenges for gay, bisexual, and other men who have sex with men (GBMSM), many of whom rely significantly on queer social spaces and sexual partnerships as sources of emotional and community support (Brennan et al., 2020; Döring, 2020). The social disruptions and additional stressors caused by the pandemic contributed to increased mental health challenges observed among GBMSM, such as heightened anxiety and depression (Philpot et al., 2021; Sanchez et al., 2020; Suen et al., 2020). These findings align closely with minority stress theory, which emphasizes how marginalized populations like GBMSM experience compounded stressors due to stigma, social exclusion, and systemic inequalities (I. H. Meyer, 2003; Mongelli et al., 2019). COVID-specific adaptations of minority stress theory further highlight how pandemic-related stressors (e.g., diminished access to in-person affirming environments and mental health supports, unsupportive home environments, etc.) exacerbated preexisting vulnerabilities among GBMSM, intensifying mental health disparities and social marginalization (Hastings & Hodge, 2023).

In response to these compounding stressors, many GBMSM turned to digital platforms to maintain social and sexual connection. Evidence suggests that during government stay-at-home orders, GBMSM continued to use gay geo-social networking applications to seek intimacy and companionship (de Sousa et al., 2020; Sanchez et al., 2020). Individual decision-making around sexual partner-seeking during the pandemic appears to have varied quite significantly, with some studies finding that GBMSM had increased their number of sexual partners since the start of the pandemic, while others found that the number of sexual partners remained the same or decreased (Gómez-Castro et al., 2022; Starks et al., 2020; Stephenson et al., 2021, 2022). These variations suggest complex sexual decision-making shaped by more than just risk assessment. Frameworks such as sexual citizenship, which emphasize the right to sexual expression, pleasure, and inclusion as fundamental components of full social participation, offer valuable lenses to understand GBMSM’s decisions during the pandemic as affirmations of identity and resistance, especially in contexts where public health discourses often pathologize queer sexual practices (Richardson, 2017).

Guided by these theoretical frameworks, this study explored GBMSM’s sexual behaviors and their comfort engaging in social and sexual venues (bars, saunas, sex parties) during the first year of the pandemic. By situating our analysis within minority stress theory and sexual citizenship perspectives, we aim to provide insight that informs culturally responsive, sex-positive, and harm reduction-oriented public health interventions in ongoing and future infectious disease outbreaks (e.g., COVID-19, mpox, HIV).

## Method

### Study Design and Participants

This study was a secondary analysis of a cross-sectional online survey conducted from October to November 2020 through *Hornet*, a global gay geo-social networking app. The survey aimed to assess the impacts of the COVID-19 pandemic on health, economic vulnerability, mental health, and HIV-related outcomes. Full study details are available elsewhere (Santos et al., 2022).

Eligible participants were active Hornet users aged 18 years or older who provided informed consent. For the present analysis, we included only cisgender men who self-identified as gay, bisexual, pansexual, asexual, or questioning. The UCLA North General Institutional Review Board reviewed the study and determined that it did not meet the definition of human subjects research under federal regulations, as it involved a secondary analysis of de-deidentified data; therefore, formal IRB approval was not required.

### Measures

#### Sociodemographic and Health Characteristics

Participants reported their age, sexual orientation, employment status, country of residence, and highest level of education. They also self-reported HIV status and history of pre-exposure prophylaxis (PrEP) use. For analysis, HIV status was recoded into three categories: HIV-negative, HIV-positive (combining those were HIV-positive and undetectable), and unknown. PrEP use was also recoded into three categories: never used PrEP, current PrEP users, and former PrEP users (collapsed from multiple former-use reasons). Participants were also asked whether their use of gay social networking apps had changed since the onset of the COVID-19 pandemic.

#### Sexual Behavior and Interest

Participants were asked how many sexual partners they had in the past month. To assess the primary outcome, they responded to the question: “Since the COVID-19 crisis began, have you had physical sexual contact with a sexual partner (date/hookup) that you met on a gay social networking app? (e.g. Hornet, Grindr, etc.)?” Response options included “yes,” “no,” “I don’t know,” and “refuse to answer.” Participants selecting the latter two were excluded from the analysis. We also asked how their interest in sex had changed since the beginning of the pandemic, measured on a 5-point Likert scale (from “strongly decreased” to strongly increased”). For analysis, we collapsed this into three categories: decreased, unchanged, or increased sex interest.

#### Comfort Attending Social and Sexual Venues

To assess comfort levels with physical venues, participants were asked when they anticipated feeling comfortable engaging in the following: 1) going to a bar; 2) attending a sauna or sex club; and 3) going to a private sex party (e.g., in someone’s home). Response options were: 1) “I feel comfortable now,” 2) “I will feel comfortable once public health officials indicate it is safe,” 3) “I will feel comfortable once there is a COVID-19 vaccine,” and 4) “I will feel comfortable once my friends start going.” Those who indicated they do not attend such venues, selected “I don’t know,” or refused to answer were excluded from analysis involving that venue.

#### National COVID-19 Stringency Level

National COVID-19 policy stringency was obtained from the Oxford COVID-19 Government Response Tracker (Hale et al., 2021), a composite index summarizing containment and closure policies such as school and workplace closures, travel bans, and stay-at-home orders. Scores were averaged across the study period, and for sensitivity analyses, the index was categorized into low, medium, and high groups based on tertiles of the mean stringency distribution.

### Statistical Analyses

We conducted three separate logistic regression models using the binary outcome of having had an app-based sexual partner since the beginning of the COVID-19 pandemic (yes/no). Each model examined one type of venue comfort (bars, saunas/sex clubs, or private sex parties) as the key predictor. This approach reduced missing data due to participants skipping questions about venues they did not frequent. All models were adjusted for potential confounders, including age, sexual orientation, employment status, and education. We also included HIV status and PrEP use as covariates in all models to examine how ongoing engagement with HIV-related care may shape sexual behavior during the pandemic. Interaction terms between venue comfort and HIV status or PrEP use were tested and found to be non-significant and therefore excluded from the final models. As a sensitivity analysis, models were re-estimated including the national COVID-19 stringency variable to account for contextual variation in government-imposed restrictions. Statistical analyses were conducted using RStudio Version 2021.09.0 (RStudio Team, 2020).

## Results

### Sample Characteristics

Participants included in this analysis (*n* = 15,499) had an average age of 35.3 years (standard deviation = 11.2 years) and represented 132 countries globally. Over three fourths of participants identified as gay (*n* = 11,808, 76.2%), 18.1% identified as bisexual (*n* = 2,809), and 5.7% identified as other sexual identities (*N* = 882, see Table 1). Over half of the sample reported having physical sexual contact with someone they met on a gay social networking app since the COVID-19 crisis began (64.8%). Additional sociodemographic information can be found in Table 1.

Over a third of participants reported spending more time on dating/hookup apps since COVID-19 (38.4%) and about a quarter had an increased interest in sex (26.4%). Most of the sample was HIV negative (71.8%) and had never taken PrEP (87.3%). Further detail on sexual health characteristics can be found in Table 2.

### Logistic Regression Analysis

Table 3 summarizes regression output for models examining social and sexual venue attendance and having app-based sex partners. Relative to individuals who reported feeling comfortable going to bars at the time the survey was conducted, those who would wait until public official indicated it was safe were 43% less likely to report sex with an app-based partner (aOR 0.57, 95% CI 0.49–0.65), while this was 55% less likely for those waiting for a vaccine (aOR 0.45, 95% CI 0.39–0.52), and 35% less likely for those waiting for their friends to start going (aOR 0.65, 95% CI 0.50–0.84). Relative to individuals who reported feeling comfortable going to saunas or sex clubs at the time of the survey, those who would wait until public official indicated it was safe were 53% less likely to report sex with an app-based partner (aOR 0.47, 95% CI 0.39–0.55), while this was 62% less likely for those waiting for a vaccine (aOR 0.38, 95% CI 0.32–0.45), and 33% less likely for those waiting for their friends to start going (aOR 0.67, 95% CI 0.47–0.97). Relative to individuals who reported feeling comfortable going to a private sex party at the time of the survey, those who would wait into public official indicated it was safe were 64% less likely to report sex with an app-based partner (aOR 0.36, 95% CI 0.29–0.43), while this was 71% less likely for those waiting for a vaccine (aOR 0.29, 95% CI 0.25–0.35), and 53% less likely for those waiting for their friends to start going (aOR 0.47, 95% CI 0.34–0.66).

In adjusted models, both HIV-positive status and current PrEP use were independently associated with higher odds of reporting an app-based sex partner across all three venue types. As neither variable significantly interacted with venue-related comfort, they are presented as covariates and excluded from Table 3 for clarity. Participants living with HIV had higher odds of reporting an app-based sex partner compared to HIV-negative participants, with adjusted odds ratios ranging from 1.54 to 1.68 across the bar, sauna, and private sex party models. Similarly, current PrEP users consistently had greater odds of reporting an app-based partner compared to those who had never used PrEP, with aORs ranging from 1.89 to 2.28. Former PrEP users also had elevated odds in the bar venue model (aOR 1.54, 95% CI 1.16–2.08), though associations in other models were not statistically significant. No meaningful associations were observed for participants with unknown HIV status.

As a sensitivity analysis, models were re-estimated including a covariate for national COVID-19 stringency level (low, medium, high) based on the Oxford COVID-19 Government Response Tracker. The inclusion of this variable did not materially change the direction or significance of associations between venue-related comfort and app-based sexual partnerships across all three venue types, indicating that results were robust to differences in national COVID-19 restrictions.

## Discussion

This study described the sexual behaviors of GBMSM in the first year of the COVID-19 pandemic among a global sample with representation from 132 countries. The majority reported an app-based sexual partner since the start of the pandemic and over one third reported spending more time on gay dating apps. Additionally, approximately a quarter expressed heightened sexual interest during the pandemic. These findings suggest significant shifts in sexual behaviors among GBMSM, which have important implications for public health and how dating apps are utilized to provide important opportunities for connection, especially during times when in-person contact may make people more vulnerable to disease transmission. Other studies similarly observed patterns of initial reductions in sexual behavior among GBMSM at the pandemic’s onset (February to April 2020), followed by increases as the crisis persisted (April to June 2020), even before vaccinations became widely available (Pampati et al., 2021). These behavioral shifts may reflect a normalization of risk as initial fears diminished or individuals prioritized emotional and sexual needs amid prolonged isolation (Braksmajer & Cserni, 2021; Loosier et al., 2024).

Our study also found that GBMSM who felt comfortable attending social and sexual venues (i.e., bars, saunas, sex clubs, and private sex parties) were more likely to engage in sex with app-based partners compared to those who preferred waiting until public health officials indicated it was safe, there was a vaccine, or their friends started going. While causality cannot be determined due to the cross-sectional design, this finding provides novel insights into the relationships between comfort in physical spaces and app-based sexual interactions. Specifically, this reflects broader trends in risk perceptions and highlights the interconnectedness of digital and physical sexual contexts. App-based sexual scripts, or the socially and culturally informed norms guiding online sexual interactions, likely evolved during the pandemic. For instance, new scripts around health status disclosures, vaccination discussions, and COVID-19 safety negotiations may have emerged, influencing GBMSM’s willingness and comfort in meeting sexual partners in person (Braksmajer & Cserni, 2021; Daroya et al., 2023). Thus, our study uniquely contributes to the literature by clarifying how digital and physical sexual environments dynamically intersect during public health crises.

Our findings also emphasize the importance of understanding sexual behaviors within broader societal and contextual factors, including differences in national COVID-19 restrictions. Sensitivity analyses accounting for policy stringency confirmed that the main associations between venue comfort and app-based sexual behavior were robust across varying restriction levels. Existing research suggests that GBMSM’s sexual behavior and decision-making during the COVID-19 pandemic was shaped by fatalism from the constant uncertainty of the pandemic, pandemic fatigue, and a longing for connection (Braksmajer & Cserni, 2021; Loosier et al., 2024). While the immediate COVID-19 crisis may have subsided with the widespread availability of vaccines and lifting of restrictions, these findings remain highly relevant. The pandemic’s enduring influence continues to shape sexual behavior, risk perception, and the ways dating apps facilitate intimacy (Velasquez et al., 2024; Wang et al., 2023).

Our findings highlight the critical need for culturally responsive, sex-positive public health interventions grounded in harm reduction principles. Historically, public health interventions targeting GBMSM often emphasized risk avoidance and abstinence-based messaging, reinforcing biomedical discourses that inadvertently pathologize queer sexual practices and identities (Cruz Neto et al., 2024). Incorporating a sexual citizenship perspective allows public health strategies to move beyond risk-based narratives and instead recognize GBMSM’s sexual practices as expressions of agency, autonomy, and community belonging. Rather than promoting abstinence-only guidelines during health crises, public health interventions could proactively offer practical harm-reduction recommendations, such as suggesting safer ways to meet partners (e.g., outdoor settings, vaccination verification, symptom disclosure), or integrating messages emphasizing pleasure, consent, and sexual agency alongside safety precautions (Ismail et al., 2022; Phillips et al., 2024; Zaneva et al., 2022). Geolocation apps also played a proactive role in this space. For instance, Tinder allowed users to display COVID-19 vaccination status, and Grindr featured banners with public health information, illustrating how platforms can serve as tools for both intimacy and health promotion during crises (Garcia-Iglesias et al., 2024). These examples reflect the broader potential for technology to facilitate not just sexual connections, but also timely access to health messaging.

Additionally, participants living with HIV and those actively taking PrEP had significantly higher odds of reporting an app-based sexual partner across venue types. One possible explanation could be that individuals with ongoing engagement in HIV-related care, either through treatment or prevention, may have felt more confident navigating sexual risk during the pandemic. This aligns with previous research showing that biomedical HIV prevention strategies often shape sexual decision-making, particularly when individuals face overlapping risk contexts such as HIV and COVID-19 (Hyndman et al., 2021; Pyra et al., 2023). These trends may also reflect the evolving conditions at the time of data collection (October–November 2020), when more information about COVID-19 transmission was available and public health guidance increasingly emphasized harm reduction (e.g., masks, outdoor gatherings, symptom screening, etc.). Concurrently, barriers to accessing HIV treatment and PrEP services, which were particularly acute early in the pandemic, had begun to ease in many settings (Meyer et al., 2023), likely supporting continued engagement in sexual health care. These findings highlight the importance of public health messages that integrate biomedical prevention tools within culturally responsive, sex-positive approaches. At the same time, they also emphasize the need for inclusive messaging that reaches GBMSM not engaged in prevention services, who may face greater barriers to accurate information or support in negotiating sexual safety.

Notably, despite engaging in behaviors counter to prevailing public health guidance, many GBMSM still took part in risk reduction strategies such as avoiding new sexual partners and opting for outdoor sex, mirroring negotiated safety strategies utilized to mitigate HIV risk (Daroya et al., 2023; Gaspar et al., 2022). Leveraging these forms of negotiated safety also requires addressing what Gaspar et al. (2022) described as *public health morality*, a normative framework that shaped expectations of what it meant to be a “responsible” sexual actor during the pandemic and influenced how GBMSM navigated sexual and health-related decisions. Public health messaging should explicitly recognize and support these nuanced harm-reduction practices rather than perpetuating restrictive, abstinence-only narratives.

Several limitations should be considered. First, data were collected online through Hornet, a gay dating app. Thus, participants may have different sexual behaviors and comfort levels attending physical venues compared to the broader GBMSM community. App-based cultures, including regional norms, varying uptake of dating apps, and distinct digital sexual scripts, may further shape sexual behaviors and decision-making patterns observed in this study (Li & Li, 2022; Wu & Trottier, 2022). Second, although participants were drawn from a diverse international sample, our analysis presented aggregate findings without exploring potential regional variations. While models adjusted for national COVID-19 stringency to account for differences in government-imposed restrictions, this measure may not capture subnational or temporal variations in policies. Third, while our analyses accounted for HIV status, PrEP use, and age as covariates, and tested interactions with venue comfort, these subgroup effects were not the primary focus of the study. Future research could further explore these dimensions as potential moderators to better understand how intersecting identities and health experiences shape sexual health behaviors and risk navigation among GBMSM during public health crises. Finally, our data, collected in late 2020 (October to November) may not reflect present-day behaviors. Nonetheless, our findings offer valuable insights applicable to ongoing and future public health crises (e.g., mpox, emerging COVID-19 variants).

## Conclusion

Five years after the onset of the COVID-19 pandemic, shifts in sexual behavior, risk perception, and dating apps continue to influence how people engage with sexuality and social interactions. Our study offers essential insights into the evolving interplay between digital and physical sexual contexts, understanding the need for public health interventions rooted in culturally responsive harm reduction and sex-positive frameworks that balance safety with the emotional and sexual needs of affected populations. The pandemic has redefined many aspects of intimacy, requiring continued attention to how dating apps and in-person social and sexual behaviors intersect with public health concerns in our “post-pandemic” world that continues to experience infectious disease outbreaks (Rubin, 2024; Zhang et al., 2024; Zinnah et al., 2024). Recognizing GBMSM’s sexual behaviors as informed by complex scripts involving safety, intimacy, and agency provides a robust basis for more inclusive and affirming public health strategies during ongoing and future health crises.

## Figures and Tables

**Table 1.** Participant Sociodemographics.

Sociodemographics	Mean (SD)	Range	N (%)
**Age (*n* = 15,499)**	35.3 (11.2)	18–85	
**Sexual Orientation (*n* = 15,499)**			
Gay			11,808 (76.2)
Bisexual			2,809 (18.1)
Pansexual			287 (1.9)
Queer			147 (0.9)
Asexual			176 (1.1)
Questioning			272 (1.8)
**Employment Status (*n* = 9,708)**			
Full-time			5,766 (59.4)
Part-time			908 (9.4)
Paid leave			150 (1.5)
Unemployed due to COVID-19			765 (7.9)
Unemployed prior to COVID-19			478 (4.9)
Student			706 (7.3)
Retired			309 (3.2)
Disability			69 (0.7)
Other			557 (5.7)
**Educational Attainment (*n* = 9,971)**			
No formal education			88 (0.9)
Elementary or primary school			128 (1.3)
High school or secondary school			1,470 (14.7)
Vocational school			803 (8.1)
Postsecondary education or some college			1,124 (11.3)
University, first degree			4,282 (42.9)
University, masters/doctorate/post-doc			2,076 (20.8)

**Table 2.** Participant sexual health characteristics.

	Mean (SD)	Range	N (%)
**Past Month Sex Partners**	2.42 (5.13)	0–110	–
**App Time since COVID-19**			
Less time			2,485 (17.4)
About the same			6,337 (44.3)
More time			5,496 (38.4)
**Sex Interest since COVID-19**			
Decreased			2,205 (19.7)
Unchanged			6,025 (53.9)
Increased			2,958 (26.4)
**HIV Status**			
HIV negative			6,744 (71.8)
HIV positive			585 (6.2)
HIV positive and undetectable			783 (8.3)
Unknown status			1,282 (13.6)
**PrEP Status**			
Never taken			8,214 (87.3)
Currently taking			789 (8.4)
Discontinued because of COVID			132 (1.4)
Discontinued for other reasons			276 (3.0)

**Table 3.** Logistic regression social and sexual venues.

	Unadjusted Odds Ratio	95% CI	Adjusted Odds Ratio	95% CI
**Comfort Bars (ref: comfort now)**	–	–	–	–
Once public health officials indicate it’s safe	0.57	**[0.50, 0.64]**	0.57	**[0.49, 0.65]**
Once there’s a vaccine	0.46	**[0.41, 0.52]**	0.45	**[0.39, 0.52]**
Once my friends start going	0.68	**[0.50, 0.64]**	0.65	**[0.50, 0.86]**
**Comfort Sauna (ref: comfort now)**	–	–	–	–
Once public health officials indicate it’s safe	0.49	**[0.42, 0.57]**	0.47	**[0.39, 0.55]**
Once there’s a vaccine	0.40	**[0.35, 0.46]**	0.38	**[0.32, 0.45]**
Once my friends start going	0.70	**[0.52, 0.95]**	0.67	**[0.47, 0.97]**
**Comfort Sex Party (ref: comfort now)**	–	–	–	–
Once public health officials indicate it’s safe	0.37	**[0.32, 0.44]**	0.36	**[0.29, 0.43]**
Once there’s a vaccine	0.32	**[0.28, 0.37]**	0.29	**[0.25, 0.35]**
Once my friends start going	0.47	**[0.36, 0.61]**	0.47	**[0.34, 0.66]**

Note: all models were adjusted for age, sexual orientation, employment status, and education.

## Data Availability

The data that support the findings of this study are available from the corresponding author, JCJ, upon reasonable request.
